# Inhibition of STAT3 blocks protein synthesis and tumor metastasis in osteosarcoma cells

**DOI:** 10.1186/s13046-018-0914-0

**Published:** 2018-10-04

**Authors:** Dongqing Zuo, Kristen L Shogren, Jie Zang, Donna E Jewison, Brian E Waletzki, Alan L Miller, Scott H Okuno, Zhengdong Cai, Michael J Yaszemski, Avudaiappan Maran

**Affiliations:** 1 Department of Orthopedic Surgery, 2-69 Medical Sciences, Mayo Clinic, 200 First St SW, Rochester, MN 55905 USA; 2 Division of Medical Oncology, Mayo Clinic, Rochester, MN USA; 3 Department of Orthopedics, Shanghai General Hospital, Shanghai Jiao Tong University, Shanghai, China; 4 Musculoskeletal Tumor Center, People’s Hospital, Peking University, Beijing, 100044 China

**Keywords:** Apoptosis, Napabucasin, Osteosarcoma, Protein synthesis, eIF4E, 4EBP-1

## Abstract

**Background:**

Osteosarcoma is the most common bone cancer. Despite advances, molecular mechanisms associated with osteosarcoma have not been fully understood. Hence, an effective treatment for osteosarcoma has yet to be developed. Even though signal transducer and activator of transcription3 (STAT3) has been implicated, its role in pathogenesis of osteosarcoma is not fully determined. In this study, we investigated the antitumor effect of napabucasin (NP) (BBI608), an inhibitor of STAT3 on osteosarcoma in vitro and in vivo and studied the underlying molecular mechanism.

**Methods:**

Cell viability, colony formation, apoptosis, tumor growth and metastasis assays were performed to examine the effect of NP on osteosarcoma in vitro and in vivo. Real-time RT-PCR, western analysis, immunofluorescence and reporter assays were used to monitor the expression and activity of proteins and underlying molecular pathways. Protein synthesis, co-immunoprecipitation and CAP binding assays were carried out to understand NP-mediated mechanism of actions in osteosarcoma cells.

**Results:**

Our results show that NP treatment decreases cell viability and induces apoptosis in several osteosarcoma cell lines. NP treatment suppresses both expression and phosphorylation of STAT3 in addition to blocking STAT3-mediated transcription and downstream target proteins in osteosarcoma cells. Furthermore, NP inhibits protein synthesis through regulation of the eukaryotic initiation factor 4E (eIF4E) and eIF4E-binding protein 1 (4E-BP1). NP also inhibits the progression of osteosarcoma tumors and metastasis in vivo in an orthotopic tibial model of osteosarcoma.

**Conclusions:**

Taken together, our investigation reveals that NP acts through a novel mechanism and inhibits osteosarcoma growth and metastasis, and could be investigated clinically for treating osteosarcoma patients alone or in combination with other drugs.

## Background

Osteosarcoma is a malignant bone tumor that affects children and young adults. Although it is considered a pediatric cancer, osteosarcoma has been known to affect adults and may be associated with other pathologic bone conditions. The standard clinical treatment in most countries includes presurgical chemotherapy, followed by surgical removal of the primary tumor after the second or third cycle of a year-long chemotherapy regimen [1–4]. A combination of surgery and chemotherapy has led to an improved survival rate in recent decades, yet about one-third of patients diagnosed with osteosarcoma develop metastatic diseases, and the survival rate is still low [2, 4–6]. The patient prognosis is often poor, as several commonly used drugs show only modest response rates. Hence, it is vital to explore new avenues for effectively targeting and treating osteosarcoma.

STAT3 is a DNA-binding protein that belongs to the signal transducer and activator of transcription (STAT) protein family, whose biologic activities regulate several functions, including cell growth, survival, and differentiation in many cell types [7–11]. Like other STAT proteins, STAT3 is activated when cells are exposed to cytokines and growth factors. After activation, STAT3 translocates into the cell nucleus from cytoplasm and binds to a specific sequence with target gene promoters to regulate gene transcription. Following the demonstration of constitutive activation of STAT3 in neoplastic cells, pharmacologic inhibition of STAT3 was investigated in several malignancies, including osteosarcoma [7, 8, 12]. Napabucasin (BBI608) (NP) is a small molecule that has been shown to block STAT3 and stemness in non-bone cancer cells [13–15]. As an attempt to develop alternative treatment, in this report we have studied the effect and molecular mechanism of action of NP on osteosarcoma in vitro and in vivo.

## Methods

### Cell lines and culture

Human osteosarcoma cell line 143B, MG63, KHOS, U2OS was obtained from the American Type Culture Collection (ATCC). Human multidrug-resistant cell line U2OSR is a kind gift from Dr. Zhenfeng Duan’s laboratory (University of California, Los Angeles, CA, USA). Cells were cultured in DMEM/F12 medium and maintained at 37 °C [16, 17].

### Drugs and antibodies

NP (99.9% purity) was purchased from Abcam (Cambridge, MA). NP was dissolved in dimethyl sulfoxide (DMSO) as a 2-mM working stock solution and stored from light in an aliquot package at − 20 °C. The working concentrations used for experiments were prepared by diluting the stock solution with DMEM/F12 medium. The following antibodies were used for Western blot analysis: GAPDH, caspase-3, survivin, Bcl-xl, PARP, Bax, c-Myc, STAT3, phospho-STAT3^Tyr705^, phospho-STAT3^Ser727^, eIF4E, phospho-4E-BP1^Thr37/46^, 4E-BP1, and non-phospho-4E-BP1 (Cell Signaling Technology, Danvers, MA, USA).

### Cell viability assay

The human osteosarcoma cells were plated at a density of 5 × 10^4^ cells per well in 24-well plates containing 1 mL/well medium. After allowing the cells to attach overnight, they were treated with different drugs or transfected with plasmid for 24, 48, and 72 h. At each end of treatment or transfection, the cell viability was determined by MTS assays as described in the manufacturer’s protocol (Promega, Madison, WI, USA).

### Cell Colony formation assay

Cells seeded in 6-well plates (500/well) were treated with a control vehicle or NP (0.3 and 0.5 μM) for 14 days. Then cells were washed with phosphate-buffered saline (PBS), fixed with 4% paraformaldehyde, and stained with 0.1% crystal violet for 15 min. The colonies with more than 50 cells were counted under a microscope.

### RNA analysis

Total RNA was extracted from Veh and NP (3.0) μM –treated cells and isolated using trizol reagent (Invitrogen), and the mRNA levels of STAT3 and the control glyceroldehyde 3-phosphate dehydrogenase (GAPDH) were analyzed by the quantitative polymerase chain reaction (PCR) as described [18].The following primer sequences were used for PCR analysis of mRNAs: STAT3 forward, 5’-GGAGGAGTTGCAGCAAAAAG-3′, STAT3 reverse, 5’-TGTGTTTGTGCCCAGAATGT-3′; GAPDH forward, 5’-ATGTTCGTCATGGGTGTGAA-3′; GAPDH reverse, 5’-TGTGGTCATGAGTCCTTCCA-3.

### Preparation and analysis of cytoplasmic extract

Cell lysis and preparation of cytoplasmic protein extract were carried out as described by 2 reports [16, 19]. Briefly, cells were resuspended with lysis buffer (0.15 M NaCl, 5 mM EDTA, 10 mM Tris-Cl, 1% Triton X-100), and protein concentrations were determined by Bradford assay. Equivalent amounts of total protein (60 μg) were electrophoretically separated with 10% or 15% polyacrylamide gel and transferred to a polyvinylidene fluoride (PVDF) filter membrane. Membranes were analyzed by Western blot hybridization using primary test antibodies and control anti-glyceroldehyde 3-phosphate dehydrogenase (GAPDH) antibodies.

Immunoprecipitation analysis was carried out as described by Kennedy and colleagues [20]. Cytoplasmic protein extracts containing 60 mg of protein were immunoprecipitated with anti-4E-BP1 antibodies and analyzed by Western blot using anti-eIF4E antibodies. The quantitation of protein signals was done using densitometer and Quantity One 4.5.2 software (BioRad, Hercules, CA, USA).

### Immunofluorescence assay

Osteosarcoma cells were grown on coverslips and treated with vehicle or NP for 12 h and fixed with 4% paraformaldehyde. They were then permeabilized with 0.1% Triton X-100 in PBS. Samples were blocked with 1% bovine serum albumin for 30 min, followed by incubation with indicated primary antibodies at 4 °C overnight. After 3 washes with PBS, cells were probed with Alexa Fluor 488 secondary antibody (ThermoFisher Scientific, Waltham, MA, USA) for 1 h at room temperature. The nuclei were stained by 4′, 6-diamidino-2-phenylindole (DAPI) and images were acquired with a fluorescence microscope.

### Hoechst 33258 assay

The Hoechst assay was used to evaluate apoptotic cell death after NP treatment. 143B and MG63 cells were seeded on coverslips at a density of 2.5 × 10^5^ cells per well in 6-well plates and exposed to vehicle or NP (3.0 μM) for 24 h. Hoechst staining and assays were carried out as described by previously [19].

### Flow Cytometry

Osteosarcoma cells were cultured in 6-well plates (2.5 × 10^5^/well) and treated as indicated for 24 h. Cell apoptosis was assayed using the Annexin V-FITC apoptosis detection kit (BD Biosciences, San Jose, CA, USA). Dual-parameter dot plots combining Annexin V-FITC/PI revealed live cells (Annexin V–/PI–), early apoptotic cells (Annexin V+/PI–), late apoptotic cells (Annexin V+/PI+), and necrotic cells (Annexin V–/PI+), respectively, in the lower left, lower right, upper right, and upper left quadrants.

### Gamma-activated sequence luciferase reporter gene assay

Cells were plated at a density of 8 × 10^4^ cells per well in 12-well plates and left overnight to settle. The next day, cells were transfected using FuGene (Promega, Madison, WI, USA) with 4 μg of gamma activated sequence (GAS) reporter plasmid DNA according to manufacturer protocol. After 24 h of transfection, cells were treated with vehicle, interferon (IFN)-γ (2,000 U/ml), and NP (3.0 μM) for 24 h. At the end of treatment, the cells were harvested and assayed for luciferase activity as described (Luciferase Assay kit, Promega). Control plasmids containing renilla luciferase were used to normalize luciferase units.

### Protein degradation assay

Osteosarcoma cells were plated and 24 h later, 2 μCi of ^14^C-L-valine was added and maintained for 24 h. Cells were then rinsed and treated with vehicle and 10 μM NP in fresh media. The media were collected at different time points. The cells were lysed, and radioactivity was determined by precipitating with 10% trichloroacetic acid and measured in a scintillation counter. The protein degradation was determined by calculating the ratio of label in the media to the cells.

### Protein synthesis (^3^H-Leucine incorporation)

The rate of protein synthesis was determined as described by Constantinou and Clemens [21]. Briefly, following vehicle and NP treatment and pulse, labeling of osteosarcoma cells was carried out for 1 h with 10 mCi/mL of radiolabeled leucine. The cells were harvested, lysed, and precipitated with 10% trichloroacetic acid (TCA). The radioactivity was measured by scintillation counting.

### Cap-binding assay

Cap-binding assay by m7GTP Sepharose chromatography was performed using osteosarcoma cell lysate (100 μg protein) and 20 mL of packed m7GTP Sepharose beads to capture eukaryotic initiation factor 4E (eIF4E) and its binding proteins as previously described [22] .

### Nude mouse tibia Orthotopic tumor model

All animal procedures were performed in accordance with a protocol approved by the Institutional Animal Care and Use Committee (IACUC) at Mayo Clinic. Four-week-old female BABL/c nude mice were used for this study, and 143B osteosarcoma cells (1.5 × 10^6^) in 20 μL of PBS was injected into the right tibial medullary cavity using a 25-gauge needle and a 100-μL syringe to establish an orthotopic osteosarcoma in vivo model. Two weeks after injection of tumor cells, the mice were randomly allocated to the vehicle (DMSO) group (*n* = 10), 10 mg/kg NP group (*n* = 10), and 20 mg/kg NP group (n = 10). Each mouse in the 2 NP groups was given an NP intraperitoneal injection every 3 days. The vehicle-group mice were injected with 100 μL of PBS with 10% DMSO the same way. The tumor volume and body weight of the animals were measured before injection using the formula: tumor volume = (length × width^2^) 0.5. Following 8 continuous injections, the mice were euthanized. Tumor legs were dissected and stored in liquid nitrogen or fixed in formalin for analysis.

Lung tissues were harvested, fixed in 10% formalin, and embedded in paraffin. Metastases in the lung were examined by gross observation and histologic assessment, as shown earlier [23]. Briefly, visible macrometastases (tumor nodules) were counted and measured with the aid of a dissecting microscope. Mice lungs were embedded in paraffin, followed by sectioning and staining with hematoxylin and eosin to detect for pulmonary metastases. Averages were determined from the total number of metastases per section. Quantity and quality of bone across the tibiae were analyzed ex vivo using Skyscan 1272 micro-computed tomography (Micro-CT) scanner (Bruker, Kontich, Belgium). Micro-CT projections were reconstructed and the bone volume was determined in 100 projections (slices) using the NRecon software (Bruker).

### Statistical analysis

Statistical analysis was performed with GraphPad Prism 5 (La Jolla, CA, USA). All values are expressed as mean ± SE. Comparisons between groups were made using the Student *t* test and 2-way ANOVA. *P* < 0.05 was considered statistically significant.

## Results

### NP blocks osteosarcoma cell growth and Colony formation

**Fig. 1:**
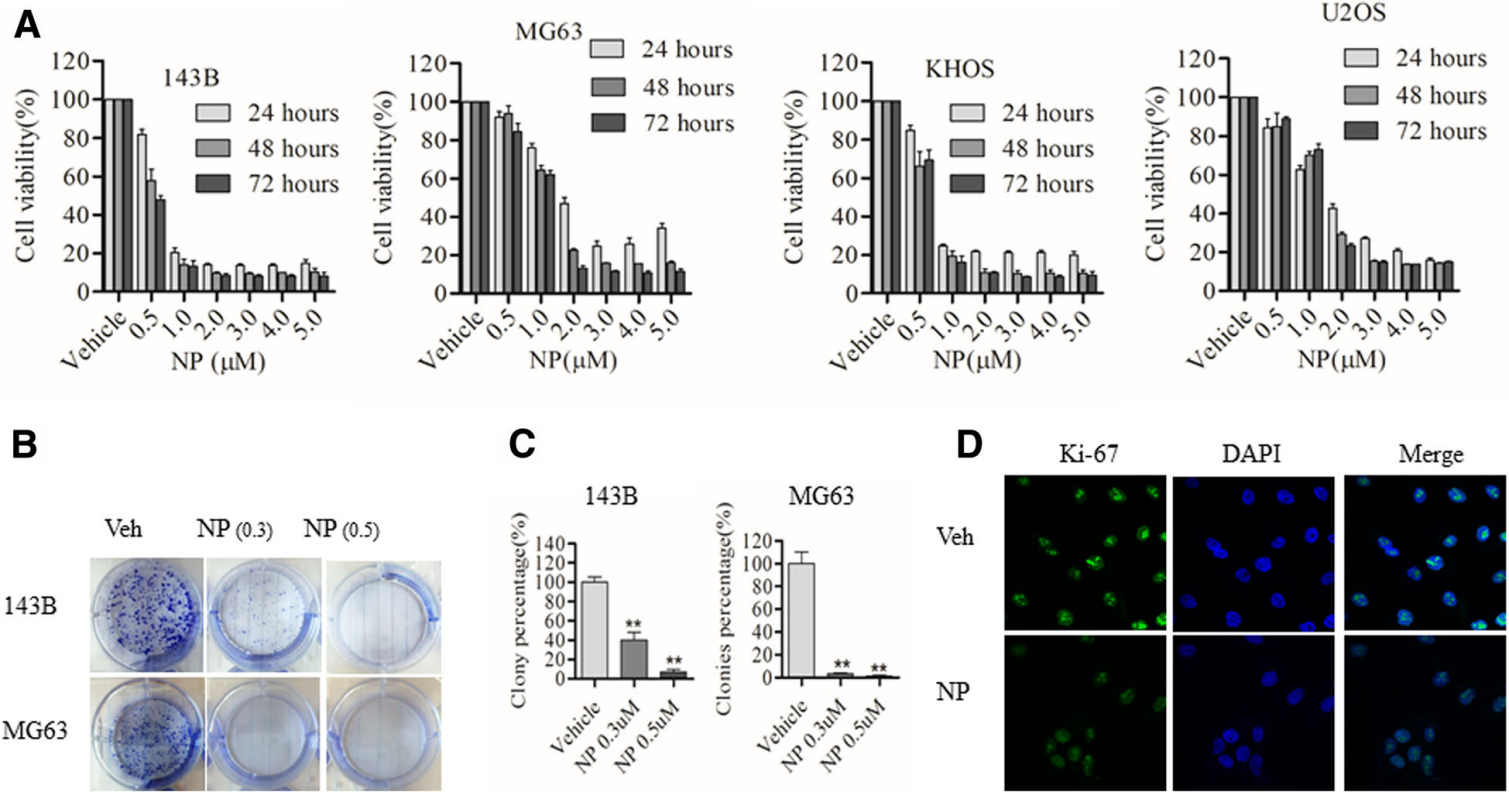
NP decreases cell viability and proliferation of human osteosarcoma cells. **a**, Human osteosarcoma cells (143B, MG63, U2OS, KHOS) were treated with vehicle (Veh) (0.1% DMSO) or NP at various concentrations for 24, 48, and 72 h, and cell viability was measured by MTS assay as described in the Methods section of the text. **b** and **c**, The cell colony formation assay was carried out in 143B and MG63 treated with Veh or NP at indicated concentrations. **d**, 143B cells were treated with Veh or NP for 24 h and analyzed by immunofluorescence using anti–Ki-67 antibodies. The data are representative of 3 independent experiments. *P < 0.05 versus vehicle control; ** *P* < 0.01 versus vehicle control

To determine whether NP blocks osteosarcoma growth, the MTS-based cell viability assay was carried out at 24 to 72 h after NP treatment in various osteosarcoma cell lines. The results show a dose-dependent effect on cell survival in several osteosarcoma cells (Fig. 1a). In the case of 143B cells, cell survival was reduced at 24, 48, and 72 h, respectively, to 84%, 52%, and 50% by 0.5 μM; to 18%, 11%, and 10% by 1 μM; to 13%, 8.9%, and 9.5% by 2 μM; to 13%, 9.2%, and 9% by 3 μM; to 12.9%, 9.8%, and 8.9% by 4 μM; and to 13%, 8%, and 9.8% by 5 μM, compared to the vehicle control. MG63 cell survival was reduced at 24, 48, and 72 h, respectively, to 78.5%, 62%, and 60% by 0.5 μM; to 50%, 23%, and 12% by 1 μM; to 22%, 16%, and 11% by 2 μM; to 29%, 15%, and 9.8% by 3 μM; to 32%, 15%, and 10% by 4 μM; and to 30%, 14%, and 10% by 5 μM, compared to the vehicle control. Similarly, the results show that KHOS cell survival was reduced at 24, 48, and 72 h, respectively, to 87%, 73%, and 74% by 0.5 μM; to 25%, 22%, and 13% by 1 μM; to 21%, 8.9%, and 11% by 2 μM; to 20.6%, 8.6%, and 9% by 3 μM; to 20%, 9%, and 9.5% by 4 μM; and to 18%, 8.8%, and 11% by 5 μM, compared to the vehicle control. In U2OS, cell survival was reduced at 24, 48, and 72 h, respectively, to 65%, 72%, and 76% by 0.5 μM; to 45%, 28.5%, and 24.6% by 1 μM; to 28%, 14.8%, and 14% by 2 μM; to 19.9%, 13.4%, and 13.8% by 3 μM; to 14.9%, 14%, and 15% by 4 μM; and to 14%, 13.5%, and 14.5% by 5 μM, compared to the vehicle control.

In order to evaluate the effect of NP on osteosarcoma cell proliferation, we carried out colony-formation assays in 143B and MG63 cells following NP treatment. We found that considerably fewer colonies were detected after treatment with 0.3 and 0.5 μM NP in 143B and MG63 cells, substantiating the inhibition of cell proliferation (Fig. 1b and c). In addition, we found that the expression of Ki-67, a cellular marker for proliferation, was suppressed following NP treatment for 24 h in 143B osteosarcoma cells (Fig. 1d).

### NP induces apoptotic cell death in human osteosarcoma cells

**Fig. 2:**
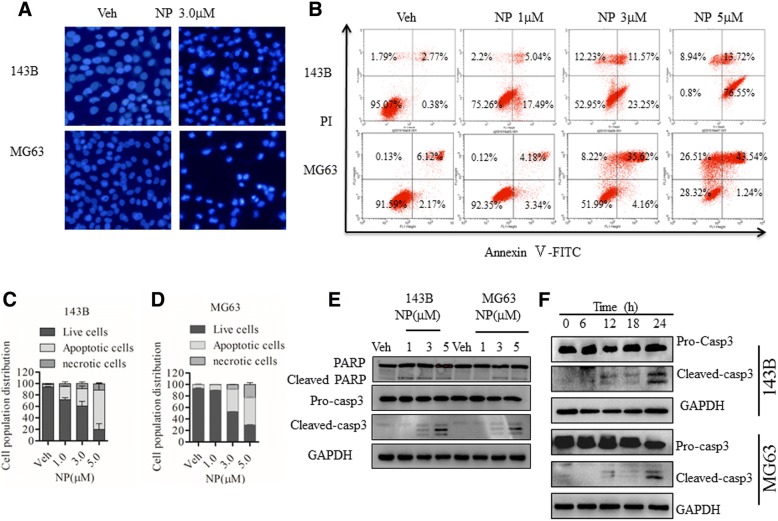
NP induces apoptosis in osteosarcoma cells in a dose-dependent manner. **a**, 143B and MG63 cells were treated with vehicle (Veh) or indicated NP (3.0 μM) for 24 h, and apoptotic morphologic changes were evaluated by fluorescence microscopy using Hoechst 33342. **b-d**, 143B and MG63 osteosarcoma cells were treated with Veh or indicated concentrations of NP for 24 h and evaluated by flow cytometry with Annexin V-FITC and PI staining. The number of apoptotic, necrotic, and live cells was quantitated. **e** and **f**, 143B and MG63 cells were treated with NP at the indicated concentrations and time. The expressions of cleaved PARP, caspase-3 were determined by Western blot using specific antibodies. The data are representative of 3 independent experiments. *P < 0.05 versus vehicle control; ** *P* < 0.01 versus vehicle control

To determine whether NP-mediated cell death was due to the induction of apoptosis, we measured apoptosis in osteosarcoma cells with Hoechst dye and Annexin V-FITC/PI staining in the presence and absence of NP treatment. Hoechst dye-positive cells increased in the presence of NP indicating apoptosis (Fig. 2a). Annexin V-FITC/PI staining analysis revealed that NP treatment at 24 h induced apoptosis in a dose-dependent manner (Fig. 2b). We further verified the induction of apoptosis by Annexin V-FITC/PI staining of cells treated with various concentrations of NP. As shown in Fig. 2b and c, NP induced apoptosis in a dose-dependent manner in both 143B and MG63 cells. At 1, 3, and 5 μM concentrations of NP, approximately 23%, 35%, and 89% of cells were apoptotic in 143B cells and 7%, 40%, and 44% of cells were apoptotic in MG63 cells, respectively (Fig. 2c and d). To further confirm these outcomes, we investigated the molecular pathways involved in the stimulation of apoptosis. As shown in Fig. 2e and f, NP activated caspase-3 and PARP cleavage in a dose- and time-dependent manner.

### NP inhibits IFN-γ–regulated STAT3 expression and activity gene induction

**Fig. 3:**
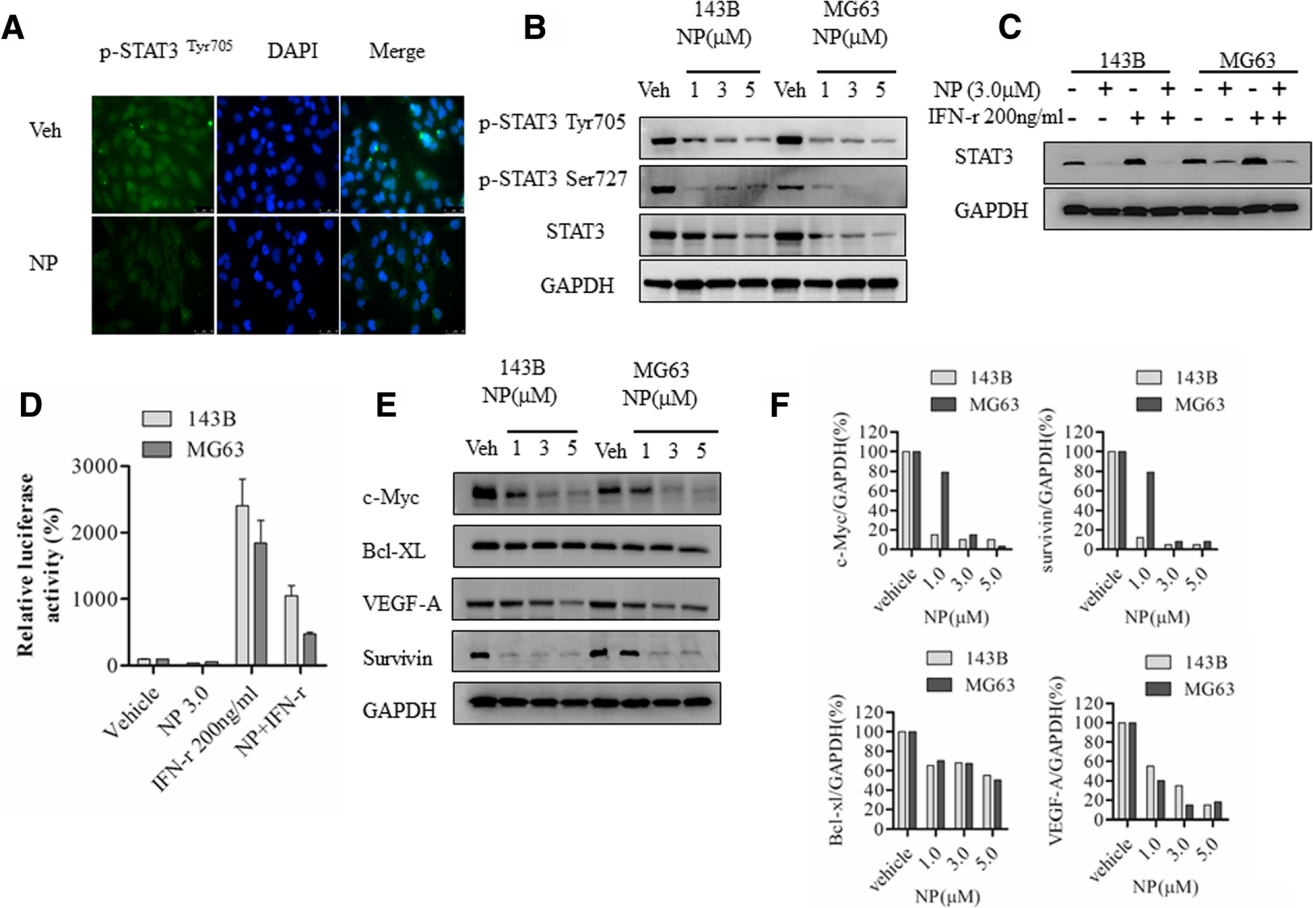
NP blocks STAT3 activation and STAT3-dependent gene expression**. a**, MG63 cells treated with vehicle or 3 μM NP for 24 h were analyzed by immunofluorescence using anti-phospho-STAT3^Tyr705^ antibodies. **b**, Cytoplasmic extracts from osteosarcoma cells treated with NP were analyzed by Western blot using anti-STAT3, anti-phospho-STAT3^Tyr705^, anti-phospho-STAT3^Ser727^, and anti-GAPDH antibodies as described in the Methods section. **c**, Cytoplasmic extracts prepared from143B and MG63 cells treated with vehicle, NP, and IFN-γ for 24 h were analyzed by Western blot analysis. **d**, 143B and MG63 cells transiently transfected with GAS-luciferase reporter plasmids were treated with vehicle and NP (3.0 μM) for 24 h, and luciferase activity was analyzed. **e**, 143B and MG63 cells were treated with vehicle or indicated concentrations of NP for 24 h. The cytoplasmic extracts prepared following the treatment were analyzed by Western blot using anti–VEGF-A, anti–c-Myc, Bcl-2, anti–Bcl-xl, anti-survivin, and anti-GAPDH antibodies. **f**, Quantitation of Western blot using densitometry. *P < 0.05 versus vehicle control; **P < 0.01 versus vehicle control

NP is known for the inhibition of STAT3 phosphorylation and activation in other cell types. Here, we evaluated the NP effect on osteosarcoma cells. Immunofluorescence in 143B osteosarcoma reveals that NP at 3 μM decreased the number of p-STAT3–positive cells; Western blot analysis showed that NP treatment markedly blocked STAT3 activation and phosphorylation of STAT3 in both Tyr^705^ and Ser^727^ sites in 143B and MG63 osteosarcoma cells (Fig. 3a and b). NP at 1, 3, and 5 μm decreased STAT3 levels in 143B cells and in MG63 cells, respectively (Fig. 3b). It has been demonstrated that IFN-γ activates STAT3 phosphorylation and STAT3-dependent transcription. To further study the effect of NP on STAT3 activation in osteosarcoma cells, we determined its effect on IFN-γ–dependent regulation and activation of STAT3. Western blot analysis showed that NP treatment blocks IFN-γ–activated induction of STAT3 expression in both 143B and MG63 osteosarcoma cells (Fig. 3c). The control GAPDH expression is not affected by IFN-γ and NP treatments (Fig. 3c). In addition, GAS luciferase reporter assays show that IFN-γ–dependent GAS luciferase activity is decreased from 25-fold to 11-fold in the presence of NP co-treatment in 143B cells (Fig. 3d). Similarly, in MG63 cells, GAS luciferase activity, which was 18-fold in the presence of IFN-γ treatment, was decreased to 5-fold when IFN-γ and NP were treated together. Our results also show that NP on its own does not have any effect on GAS-dependent luciferase activity (Fig. 3d).

STAT3 has been previously implicated in the regulation of anti- or pro-apoptotic regulators. Our results showed that c-Myc, survivin, vascular endothelial growth factor (VEGF)-A, and Bcl-xl were downregulated following NP treatment in osteosarcoma cells (Fig. 3e and f).

### NP treatment Downregulates protein synthesis initiation factors eIF4E and 4E-BP1 and blocks protein synthesis in osteosarcoma cells

**Fig. 4:**
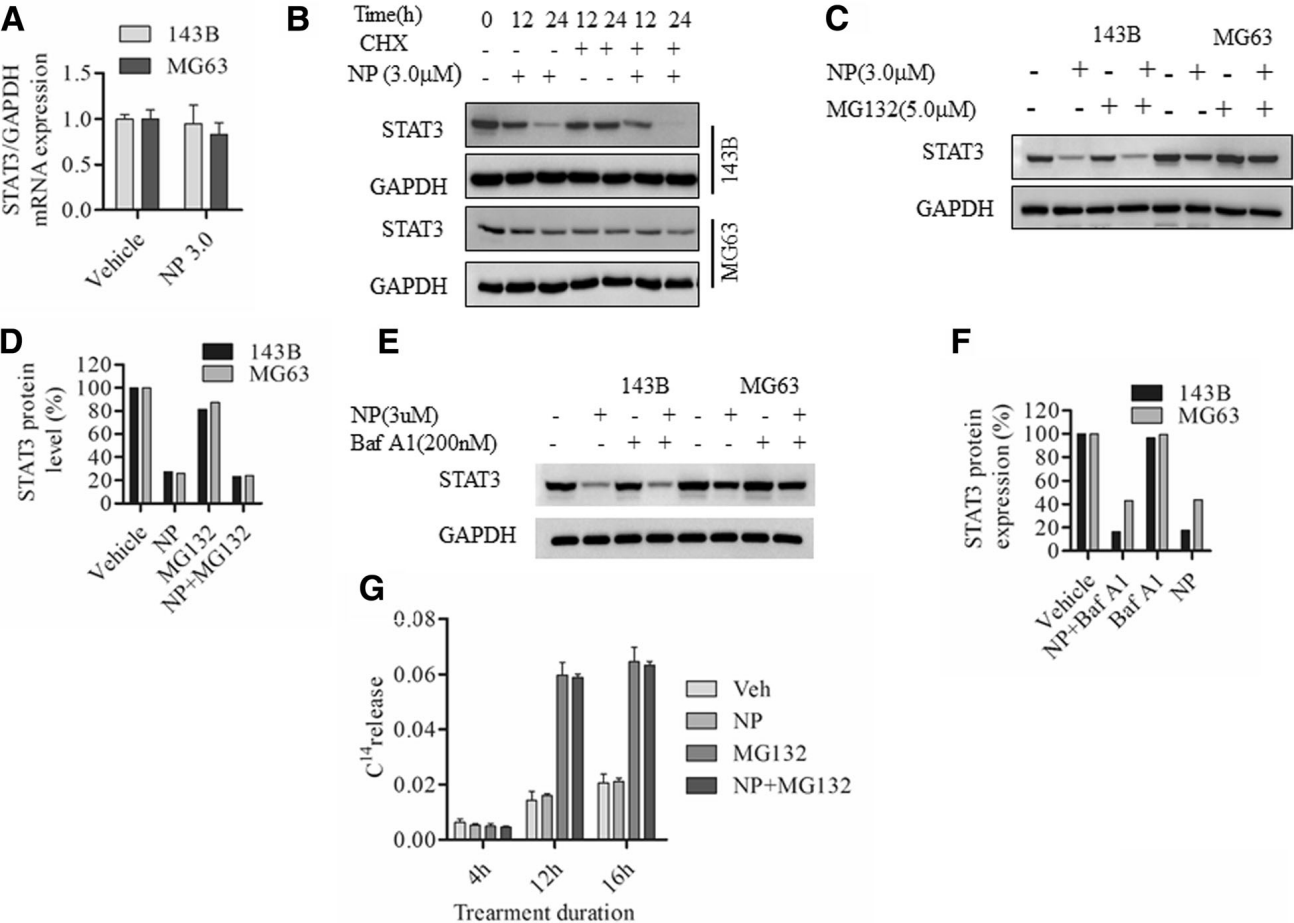
NP treatment inhibits protein synthesis in osteosarcoma cells**. a**, 143B and MG63 cells were treated with vehicle or NP (3.0 μM) for 24 h, and STAT3 mRNA expression was determined by RT-PCR. **b**, 143B and MG63 cells were treated with vehicle or NP (3.0 μM) with or without protein synthesis inhibitor cycloheximide (CHX) for 0, 12, and 24 h. Cytoplasmic extracts were prepared and analyzed by Western blot using anti-STAT3 antibodies. **c** and **d**, 143B and MG63 cells were treated with vehicle or NP (3.0 μM) in the presence and absence of proteasome inhibitor MG132 (5.0 μM) for 24 h. Cytoplasmic extracts were prepared and analyzed by Western blot using anti-STAT3 antibodies. **e** and **f**, 143B and MG63 cells were treated with vehicle or NP (3.0 μM) with or without lysosome protein degradation inhibitor Baf-A1 (200 nM) for 24 h, and cytoplasmic extracts were prepared and analyzed by Western blot using anti-STAT3 antibodies. **g**, ^14^C-valine–labeled MG63 cells were treated with vehicle and NP (3.0 μM) in the presence and absence of MG132 (5.0 μM) for 4, 12, and 16 h, and protein degradation was analyzed as described in the Methods. *P < 0.05 versus vehicle control; ** *P* < 0.01 versus vehicle control

In order to determine whether NP has a direct effect on the mRNA levels of STAT3, we carried out quantitative polymerase chain reaction (PCR). The results showed that NP treatment displays only minimal changes after 24 h, and STAT3 mRNA levels decreased by 0.95 ± 0.3-fold and 0.90 ± 0.2-fold in 143B and MG63 cells, respectively (Fig. 4a). To determine whether NP-induced changes are associated with translational changes, we investigated the effect of NP in the presence and absence of cycloheximide (CHX) in osteosarcoma cells. The results showed that NP treatment promotes CHX-mediated protein synthesis inhibition (Fig. 4b). Further, we used 2 protein degradation inhibitors to verify this finding. Our results showed that neither proteasome inhibitor MG132 (Fig. 4c and d) nor lysosome inhibitor Bafilomycin A1 (Baf-A1) (Fig. 4e and f) could restore NP-mediated STAT3 inhibition. Additional studies to investigate the effect of NP treatment on protein degradation using a ^14^C labeling showed that the NP plus MG132 treatment group displayed exactly the same ^14^C release as the MG132 treatment alone (Fig. 4g), indicating that NP actions do not involve protein degradation.

**Fig. 5:**
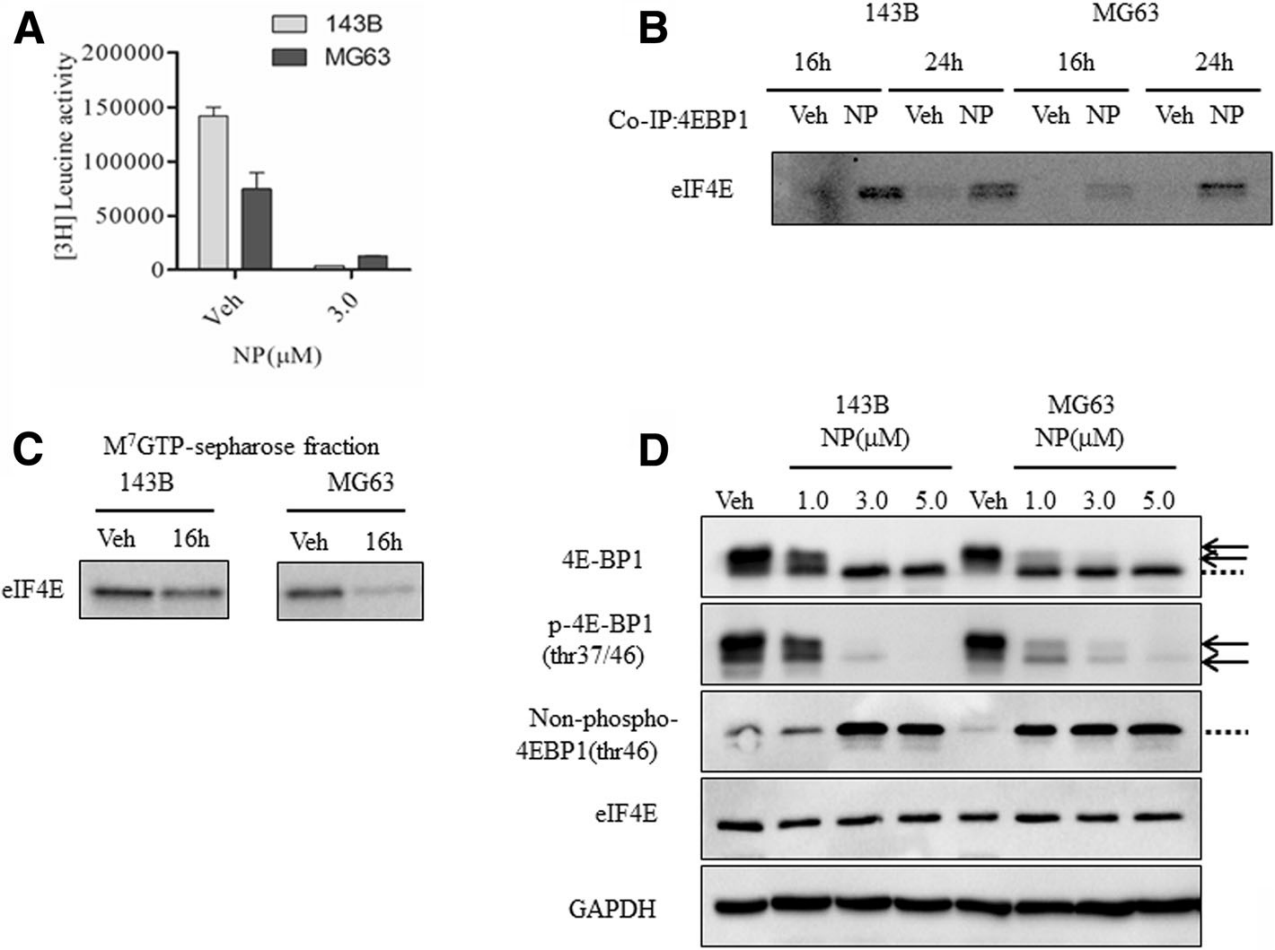
NP regulates eIF4E and 4E-BP1 functions in osteosarcoma cells**. a**, Protein synthesis was measured following vehicle and NP treatment in 143B and MG63 cells at 24 h through pulse labeling with ^3^H-leucine. **b**, Cytoplasmic extracts were prepared from 143B and MG63 osteosarcoma cells at 16 and 24 h following Veh and NP (3.0 μM) treatment. The extracts were subjected to immunoprecipitation (IP) with anti-4E-BP1 antibodies (**b**) or cap-binding (**c**) and analyzed by Western blot analysis using anti-eIF4E antibodies. **d**, Cytoplasmic extracts were analyzed by Western blot using anti–4E-BP1, anti–phosph-4EBP1Thr37/46, anti–non-phospho-4E-BP1, anti-eIF4E, and anti-GAPDH antibodies. Arrows represent phosphorylated proteins and dotted lines represent non-phosphorylated proteins

We then evaluated the effect of NP on protein synthesis in osteosarcoma cells through ^3^H-labeling studies. The results showed that the rate of protein synthesis is considerably lowered following 24 h of NP treatment in 143B and MG63 cells to 1% and 8%, respectively, compared to vehicle (Fig. 5a). Previous results showed that a decrease in protein synthesis is coupled with a block in protein synthesis at the level of translation initiation. To determine whether NP regulates protein synthesis block at the level of initiation, we investigated the regulation of eIF4E and eIF4E-binding protein 1 (4E-BP1) in osteosarcoma cells. Analysis of cytoplasmic extracts by co-immunoprecipitation studies from vehicle- and NP-treated osteosarcoma cells show that NP increased the binding of 4E-BP1 to eIF4E at 16 h in 143B and MG63 cells, respectively (Fig. 5b). Also, the cap-binding assays show that NP treatment resulted in decreased binding of eIF4E to cap structure (Fig. 5c). Western blot analysis revealed that NP does not affect the levels of eIF4E and control protein GAPDH, though it does modulate 4E-BP1 protein levels in osteosarcoma cells at the level of phosphorylation (Fig. 5d). Using specific phospho (anti-phospho-4E-BP1) and non-phospho (anti-nonphospho 4E-BP1) antibodies that recognize hypo- and hyper-phosphorylated proteins (Fig. 5d, arrows) and non-phosphorylated proteins (Fig. 5D, dotted lines) and the total antibody (anti-4E-BP1) that recognizes both the phosphorylated and non-phosphorylated forms, we demonstrated that NP increases non-phosphorylated protein levels of 4E-BP1 levels (Fig. 5d).

### NP inhibits growth of osteosarcoma in vivo

**Fig. 6:**
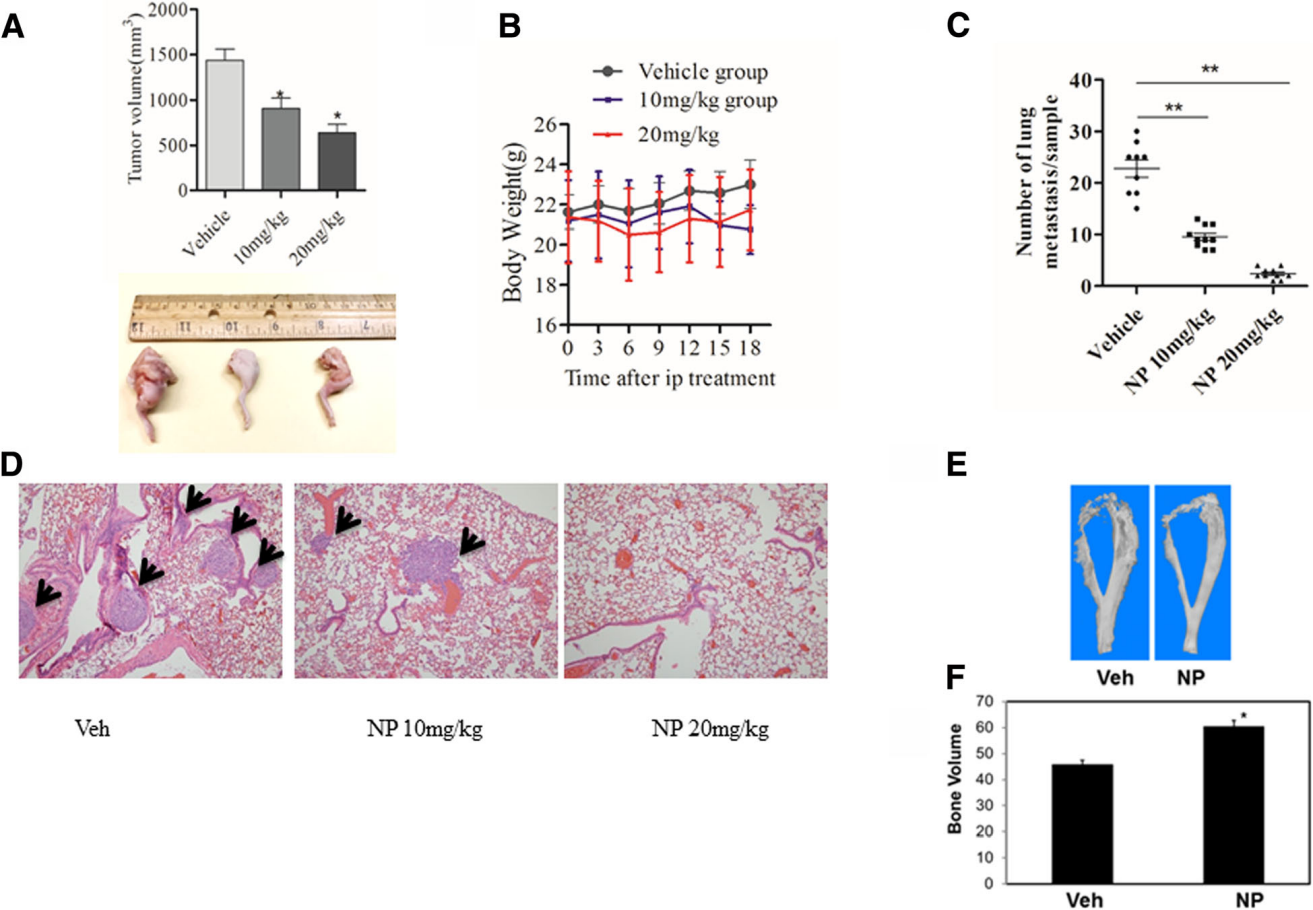
NP inhibits osteosarcoma growth in vivo**.** Measurement of tumor volume (**a**) and body weight (**b**) following Veh and NP treatment in osteosarcoma animals. **c** and **d**, Quantitation of lung metastases (shown by black arrows) and hematoxylin and eosin staining of lung tissues following Veh and NP treatments in mice with osteosarcoma. **e**, Representative micro-CT images of tibia osteosarcoma tumors in mice after treatment with Veh and NP. **f**, Bone volume measurements. *P < 0.05 versus vehicle control; **P < 0.01 versus vehicle control

The in vivo anti-tumor effect of NP was determined using nude mouse models of osteosarcoma developed through intratibial injection of osteosarcoma cells. Our results show that intraperitoneal administration of NP at doses of 10 and 20 mg/kg resulted in decreased tumor volume to 43.8% and 60%, respectively (Fig. 6a). Notably, 10 and 20 mg/kg of NP treatment induced 6% and 14% weight loss in mice, respectively, following treatment (Fig. 6b). Additionally as shown in Fig. 6c and d, NP administration inhibited osteosarcoma lung metastasis in nude mice. Hematoxylin and eosin staining and quantitation of stained lung sections revealed 60% and 80% decreases in the number of metastatic nodules in animals treated with 10 and 20 mg/kg of NP, respectively (Fig. 6c and d).

Micro-CT analysis of mouse bone specimen was used to quantify the effect of NP on bone destruction and osteolysis. The results showed bone erosion in tumor legs treated with vehicle and further showed that osteosarcoma-induced osteolysis was reduced in the bones of mice that were treated with NP (Fig. 6e). The bone volume of the distal tibia injected with NP was increased by 33%, suggesting that NP treatment in mice reduced tumor-induced bone destruction (Fig. 6e).

## Discussion

Apart from mediating downstream signaling of cytokines, growth factors, and transcriptional regulations, STAT3 proteins have been implicated in several cellular processes, including cell proliferation and tumorigenesis [7–9, 12, 24] . In this report, we showed that the STAT3 inhibitor NP induces cell death and inhibits STAT3 protein expression, phosphorylation, and transcriptional activity in osteosarcoma cells. Also, this study showed that NP-mediated regulation of STAT3 in osteosarcoma cells involves down regulation of protein synthesis initiation factors and inhibition of protein synthesis. Additionally, NP treatment blocks osteosarcoma growth in nude mice models. These results demonstrate novel downstream mechanisms of STAT3 and reveal that NP, which effectively blocks osteosarcoma growth in vitro and in vivo, could be further explored clinically in the control of osteosarcoma tumors and metastasis.

Activation of STAT3 and its role as an oncogene has been demonstrated in many tumors [8, 24]. Several studies have employed the inhibition of STAT3 as a beneficial anticancer strategy to target human tumors of both solid and hematologic origin [24]. Recently, NP has been used to target numerous malignancies. Our results show that NP blocks cell growth and decreases cell viability in several osteosarcoma cells. Furthermore, the proliferation marker Ki-67 is downregulated in NP-treated osteosarcoma cells. These observations are substantiated by decreased tumorigenicity and colony formation in the presence of NP treatment. Evaluation of the mechanism of growth inhibitory actions of NP in MG63 and 143B osteosarcoma cells suggests that NP induces apoptosis and cleavage of molecular markers, PARP, and Pro-Caspases 3. Thus, these results demonstrate that anti-tumorigenic effects of NP involve inhibition of cell proliferation and induction of apoptosis in osteosarcoma cells.

STAT3 is constitutively activated in many tumors, including osteosarcoma [24–26]. STAT3 activation can occur through phosphorylation of tyrosine 705 or serine 727. It has been demonstrated by earlier investigations that tyrosine phosphorylation contributes to translocation of STAT3 to the nucleus, activation of target genes, and tumorigenesis. In addition, reports show that phosphorylation at serine 727 is required for the highest transcriptional activity of STAT3 [27] and stimulates prostate tumorigenesis independent of tyrosine 705 phosphorylation [28]. Furthermore, sources suggest that the constitutive phosphorylation of STAT3 at serine 727 is detected in various types of human malignancies and is essential for tumor cell growth and invasion [29, 30]. Current findings show that NP regulates STAT3 at multiple levels and blocks both tyrosine and serine phosphorylation of STAT3 proteins in addition to decreasing its expression levels in osteosarcoma cells. Our results also show that cytokine (IFN-γ)–mediated activation of STAT3 activity and STAT3-dependent transcription in osteosarcoma cells are blocked by NP. Previous reports indicate that STAT3 can regulate several genes, including anti- or pro-apoptotic regulators. Our results show that c-Myc, survivin, VEGF-A, and Bcl-xl were downregulated following NP treatment in osteosarcoma cells. These observations are in agreement with previous reports in osteosarcoma and other cancers, which show that inhibition of STAT3 leads to decreased expression of survivin, VEGF-A, and Bcl-xl. Recently, Oi et al. showed that a plant-derived STAT3 inhibitor, cucurbitacin, suppresses the expression of c-myc and survivin proteins [31]. Also, siRNA-mediated inhibition of STAT3 leads to the downregulation of survivin and VEGF-A in canine osteosarcoma cells [25]. NP treatment has been shown to downregulate survivin and c-Myc in prostate cancer models [14, 15]. Thus, our current study shows that c-Myc, VEGF-A, and survivin are downregulated in osteosarcoma cells following the inhibition of STAT3 activity and confirms that molecular signaling downstream of STAT3 is blocked.

The NP-mediated effect does not require new protein synthesis, as shown by cycloheximide co-treatment. Also, it is not affected by proteasome inhibitor MG132 treatment and does not involve proteolytic degradation pathways. Our studies reveal that NP treatment leads to a block in protein synthesis in 143B and MG63 osteosarcoma cells. Previous reports showed that the regulation of protein synthesis at the initiation level plays an important role in the control of tumor cell proliferation [32–34]. The role of the protein synthesis initiation factor, eIF4E, has been studied in detail in tumor cells [32, 35]. The eIF4E is the 5′cap-binding protein that controls ribosome recruitment at the mRNA 5′ end, whose activity is inhibited by 4E-BPs (eIF4E-binding proteins). The 4E-BP regulates the eIF4E ability to form a cap-binding complex. The 4E-BPs are phosphorylated in response to cytokines, growth factors, and anti-tumor compounds [32, 35]. When 4E-BP is hypophosphorylated, it can sequestrate eIF4E and block translational initiation by blocking the interaction of eIF4E with eIF4G. The eIF4E is a rate-limiting factor of the eIF4F protein complex and is essential for all mRNAs to be translated into proteins. The protein complex binds to 7-methyl-guanosine-triphosphate cap structure in mRNAs, which further facilitates binding to ribosomes and cap-dependent protein synthesis [33, 35]. The eIF4D also requires cap binding for its ability to promote growth and to transform cells. Our results reveal an increased binding of eIF4E and 4E-BP1 and decreased binding of eIF4E to 7-methyl-guanosine cap structure in the presence of NP treatment. These observations are further confirmed by increased levels of non-phospho 4E-BP1 and decreased levels of phospho 4E-BP1 in the presence of NP treatment in osteosarcoma cells. Studies reveal that, compared to normal cells, tumor cells rely more on cap-dependent protein synthesis and synthesis of oncogenic proteins [21, 33, 35]. In addition, dysregulated transport of mRNAs of oncogenes appears to be the reason for the oncogenic functions of eIF4E. Overall, these studies indicate that NP could work, partly, through the regulation of protein synthesis. Our findings, which reveal a novel downstream regulatory mechanism for STAT3, is corroborated by the pleotropic functions of STAT3 in various cell types, contributing to transcriptional regulation, mitochondrial activities, interaction with DNA methyl transferase 1, and tumorigenesis [36, 37]. This observation is also in agreement with earlier reports regarding the role of eIF4E and 4E-BPs in osteosarcoma in vitro and in vivo. A previous report showed that eIF4E could work downstream of critical antitumor pathways [22]. Additionally, evidence points out that STAT3 could be co-regulated with eIF4E in human malignancies [38]. Thus, given the apparent role of the eIF4E pathway in malignant transformation, it is possible that NP-induced STAT3-dependent control of osteosarcoma cell functions could, in part, work through the regulation of protein synthesis. Further work is needed to understand the complete mechanisms and target gene mRNAs that are regulated at the level of protein synthesis initiation following NP treatment.

Other than standard chemotherapy, no new drug has been introduced to treat osteosarcoma in the past 2 decades. Recently, the advancement of pathway-targeted drugs has offered hope in blocking tumorigenic pathways in osteosarcoma. Our work shows that NP treatment blocks tumor growth, metastasis and tumor-induced osteolysis in animals with osteosarcoma. Thus, our work, which reveals that NP blocks STAT3-dependent tumor progression and metastasis of osteosarcoma, could be useful in targeting osteosarcoma.

## Conclusions

Our studies show that NP-mediated inhibition of STAT3 blocks osteosarcoma growth in vitro and in vivo. NP-mediated anti-tumor effects involve downstream control of protein synthesis. Taken together, our findings reveal that NP actions involve novel mechanisms in osteosarcoma, and it could be worthy of further clinical evaluation in the treatment of osteosarcoma alone or in combination with other chemotherapeutic agents.

## Abbreviations

4E-BP1: eIF4E-binding protein 1
CHX: Cycloheximide
DOX: Doxorubicin
eIF4E: Eukaryotic initiation factor 4E
GAS: Gamma activated sequence
IFN: Interferon
micro-CT: micro-computed tomography
NP: Napabucasin
STAT3: Signal transducer and activator of transcription3
